# Raising the Bar in Crohn’s Disease: Is Transmural Healing Achievable?

**DOI:** 10.3390/diagnostics16172749

**Published:** 2026-08-27

**Authors:** Fabrizio Fanizzi, Alessandra Zilli, Tommaso Lorenzo Parigi, Virginia Solitano, Sara Massironi, Federica Furfaro, Laurent Peyrin-Biroulet, Mariangela Allocca, Silvio Danese, Ferdinando D’Amico

**Affiliations:** 1 Gastroenterology and Endoscopy, IRCCS San Raffaele Hospital, 20132 Milan, Italy; 2 Faculty of Medicine and Surgery, Vita-Salute San Raffaele University, 20132 Milan, Italy; 3 Northwestern Medicine, Digestive Health Institute, Chicago, IL 60611, USA

**Keywords:** Crohn’s disease, transmural healing, treat-to-target, intestinal ultrasound, magnetic resonance enterography, cross-sectional imaging, deep remission, inflammatory bowel disease

## Abstract

**Background:** The therapeutic paradigm of Crohn’s disease (CD) has progressively evolved from symptom control toward objective treatment targets aimed at modifying disease course. Although endoscopic mucosal healing (MH) is currently regarded as a major treatment goal, it may inadequately reflect residual inflammation in a condition characterized by transmural bowel involvement. Consequently, transmural healing (TH), assessed through cross-sectional imaging modalities, has emerged as a potential marker of deeper disease control. **Methods:** A comprehensive literature review was conducted to identify studies evaluating the definition, assessment, prognostic significance, and achievability of TH in CD. Original studies, randomized controlled trials, systematic reviews, consensus statements, and international guidelines were included. **Results:** Accumulating evidence indicates that TH is associated with superior long-term outcomes compared with MH alone, including lower risks of hospitalization, surgery, treatment escalation, disease relapse, and bowel damage progression. However, substantial heterogeneity persists regarding the definition of TH across imaging modalities, with intestinal ultrasound and magnetic resonance enterography representing the principal tools for its assessment. Available data suggest that TH can be achieved in a meaningful proportion of patients receiving advanced therapies, particularly when treatment is initiated early in the disease course, although rates vary considerably according to therapeutic class, disease characteristics, and the definition adopted. Emerging technologies may further refine the assessment of transmural disease activity. **Conclusions:** TH represents a promising therapeutic target that may better capture the full burden of inflammation in CD than mucosal endpoints alone. Future efforts should focus on harmonizing imaging criteria and determining whether targeting TH can translate into meaningful disease modification and improved long-term outcomes.

## 1. Introduction

The therapeutic landscape of Crohn’s disease (CD) has undergone a remarkable evolution over the past two decades. Historically, treatment strategies were primarily focused on symptom control, corticosteroid sparing, and improvement in quality of life. However, growing evidence has demonstrated that clinical symptoms correlate poorly with underlying inflammatory activity and fail to accurately reflect the risk of progressive structural bowel damage. This recognition has led to a paradigm shift from symptom-based management toward objective monitoring and treat-to-target strategies aimed at altering the natural history of the disease [1].

The introduction of the treat-to-target concept has progressively raised therapeutic expectations in CD. Clinical remission was initially complemented by biochemical remission, followed by the adoption of endoscopic mucosal healing (MH) as a major treatment target. MH has consistently been associated with improved long-term outcomes, including reduced hospitalization rates, lower need for surgery, and sustained corticosteroid-free remission. Consequently, it has become the primary endpoint in most clinical trials and an important therapeutic goal in routine practice [2,3]. Nevertheless, whether mucosal healing represents the optimal endpoint in a disease characterized by transmural inflammation remains an open question.

Unlike ulcerative colitis, CD involves the entire bowel wall and may extend beyond the intestine to affect the mesentery and surrounding tissues. Persistent transmural inflammation may therefore exist despite apparent endoscopic remission, potentially contributing to ongoing tissue injury, fibrosis, stricturing complications, and progressive bowel damage [4]. This biological discrepancy has prompted increasing interest in therapeutic targets capable of capturing the full extent of disease activity rather than mucosal abnormalities alone. In this context, TH has emerged as a promising marker of deep disease control [1]. Enabled by advances in cross-sectional imaging, TH aims to assess resolution of inflammation throughout the bowel wall and, potentially, beyond it. A growing body of evidence suggests that patients achieving TH experience superior long-term outcomes compared with those achieving MH alone and these findings have fueled the hypothesis that TH may represent a more comprehensive measure of disease modification than conventional endoscopic endpoints [5,6,7,8]. As treatment goals in CD continue to evolve toward true disease modification, a critical question emerges: should transmural healing be considered the next therapeutic target? In this review, we critically examine the rationale for TH in CD, discuss current definitions and imaging approaches, evaluate the evidence linking TH to long-term outcomes, and explore whether achieving transmural healing is both realistic and clinically meaningful in the era of advanced therapies.

## 2. Materials and Methods

This narrative review was based on a structured literature search conducted in PubMed, Embase, and Scopus to identify studies evaluating TH in CD. The search included articles published up to 31 May 2026, with additional manual searches performed to identify recently published relevant studies.

The search strategy combined terms related to TH and cross-sectional imaging, including “transmural healing”, “transmural remission”, “transmural response”, “Crohn’s disease”, “intestinal ultrasound”, “IUS”, “magnetic resonance enterography”, “MRE”, “computed tomography enterography”, “CTE”, “mucosal healing”, “deep remission”, and “treat-to-target”. Only articles published in English were considered.

Titles and abstracts were screened independently by two reviewers, followed by full-text assessment of potentially relevant articles. Original studies, randomized controlled trials, systematic reviews, meta-analyses, consensus statements, and international guidelines were considered. Reference lists of selected publications were also manually reviewed to identify additional relevant studies. Studies were selected based on their relevance to the objectives of this narrative review, namely the definition, assessment, prognostic significance, achievability, and potential role of TH as a treatment target in CD. As this was a narrative review rather than a systematic review or meta-analysis, no formal PRISMA-guided study selection process or flow diagram was applied.

## 3. Beyond Mucosal Healing: The Rationale for Transmural Healing

CD is characterized by transmural inflammation that may persist despite apparent endoscopic remission. Although MH has become a major therapeutic target and is associated with improved clinical outcomes, endoscopy evaluates only the luminal surface and may not fully capture ongoing bowel wall inflammation [1]. This discrepancy has raised concerns that MH alone may underestimate residual disease activity and the risk of progressive bowel damage. More importantly, this discrepancy appears to have relevant prognostic implications. Several studies have demonstrated that patients achieving TH experience more favorable long-term outcomes than those achieving MH alone [5,6,7,8,9]. The prospective cohort by Castiglione et al. provided some of the most compelling evidence. In 218 patients with CD treated with anti-TNF agents for two years, TH, defined as a bowel wall thickness (BWT) ≤ 3 mm, was achieved in 31.2% of cases and was associated with a clear gradient of clinical outcomes according to the depth of healing achieved. Patients reaching TH showed the highest rates of steroid-free clinical remission at one year (95.6%), together with the lowest rates of hospitalization (8.8%) and surgery (0%), compared with those achieving MH alone (75%, 28.3%, and 10%, respectively) or no healing (41%, 66.6%, and 35.5%, respectively; *p* < 0.001). Importantly, TH was also associated with longer intervals free from clinical relapse, hospitalization, and surgery compared with MH, and retained its prognostic advantage even among patients who discontinued biologic therapy [9].

Similar findings were subsequently reported by Fernandes et al. in a cohort of 214 patients undergoing paired magnetic resonance enterography (MRE) and colonoscopic assessment. Patients achieving TH showed remarkably low rates of hospitalization (3%), treatment escalation (15.2%), and surgery (0%), supporting the hypothesis that resolution of transmural inflammation may provide a more durable disease control than endoscopic remission alone [5].

This concept was further reinforced by studies directly comparing different healing states. In a large real-world cohort of 392 patients receiving anti-TNF therapy, Oh et al. classified patients according to endoscopic healing, radiologic healing, combined endoscopic and radiologic healing (“deep healing”), or no healing. Patients achieving deep healing showed the most favorable long-term prognosis, whereas those achieving only endoscopic or only radiologic healing experienced significantly higher risks of adverse outcomes. In multivariable analysis, endoscopic healing alone (aHR 3.90, 95% CI 1.64–9.32) and radiologic healing alone (aHR 3.84, 95% CI 1.55–9.56) were both associated with a substantially greater risk of major adverse outcomes compared with deep healing, while patients with no healing exhibited the highest risk (aHR 8.84, 95% CI 4.27–18.32). These findings suggest that neither mucosal nor radiologic remission alone fully captures the depth of disease control achieved when both compartments are simultaneously healed [6]. Further evidence came from the study by Buisson et al., in which MRI-defined bowel wall healing independently predicted sustained corticosteroid-free remission (OR 4.42, 95% CI 2.29–26.54) and a significantly lower risk of CD-related surgery (HR 0.16, 95% CI 0.043–0.63) [7]. The superiority of transmural targets over mucosal endpoints was further demonstrated by Lafeuille et al., who evaluated 154 patients undergoing paired MRI and endoscopic assessment. Interestingly, MRI healing alone and complete TH were both associated with a significantly lower risk of bowel damage progression than MH alone (HR 0.09 and HR 0.05, respectively). Moreover, both imaging-based endpoints were associated with fewer major adverse outcomes, including hospitalization, surgery, and treatment discontinuation. Notably, the strongest protective effect was observed among patients achieving complete TH [8]. More recently, Revés et al. further confirmed the prognostic value of TH in a multicenter MRE-based cohort. Independent of treatment timing and previous biologic exposure, patients achieving TH experienced significantly lower risks of bowel damage progression (aHR 0.28, 95% CI 0.10–0.79), CD-related surgery (aHR 0.21, 95% CI 0.05–0.88), and treatment escalation (aHR 0.35, 95% CI 0.14–0.88) [10].

Collectively, these observations suggest that the value of TH extends beyond radiological normalization and may reflect a deeper level of disease control than that captured by endoscopy alone. In a disease fundamentally defined by transmural inflammation and progressive bowel damage, therapeutic targets capable of assessing the entire bowel wall may provide a more meaningful measure of disease modification than mucosal endpoints alone. This concept provides the biological and clinical rationale for the growing interest in TH as a treatment target in CD [1,11].

As therapeutic targets continue to evolve, additional concepts including histological healing and deep healing have been introduced. Histological healing has gained increasing attention in ulcerative colitis as a component of the broader concept of disease clearance, a multidimensional therapeutic target integrating clinical, endoscopic, and histological remission. In CD, however, the role of histological healing remains less clearly established because biopsy specimens assess only the superficial mucosal layer and cannot capture persistent transmural inflammation [12]. Likewise, deep healing has been used to describe composite endpoints combining both MH and TH, although its definition varies considerably across studies [13]. These concepts should therefore be regarded as complementary rather than interchangeable with TH, which specifically reflects normalization of bowel wall inflammation assessed by cross-sectional imaging in CD.

### Current Guideline Position of Transmural Healing

Current international guidelines recognize the growing value of cross-sectional imaging in the assessment and monitoring of CD but do not currently recommend TH as a formal treatment target. The STRIDE-II recommendations identify clinical remission, normalization of biomarkers, and endoscopic healing as the primary therapeutic targets, while TH is acknowledged as an important adjunctive measure requiring further validation before incorporation into treat-to-target algorithms [1]. Similarly, recent ECCO-ESGAR-ESP-IBUS guidance emphasizes the complementary role of IUS and MRE for disease monitoring. Therefore, current recommendations position TH as a promising research endpoint and a potential future therapeutic target rather than a standard goal of current routine clinical practice [14,15].

## 4. Current Definitions and Assessment of Transmural Healing

The concept of TH has evolved alongside the increasing use of cross-sectional imaging in CD. Unlike histological healing or the broader concept of deep healing, TH specifically refers to the resolution of inflammation throughout the entire bowel wall as assessed by cross-sectional imaging. Early studies referred broadly to “radiologic” or “imaging” healing, generally describing the absence of inflammatory abnormalities on MRE, computed tomography enterography (CTE), or intestinal ultrasound (IUS). Over time, accumulating evidence linking imaging remission to improved clinical outcomes led to the development of more specific definitions aimed at capturing complete resolution of bowel wall inflammation. However, TH still lacks a universally accepted definition [11]. Considerable heterogeneity exists among studies regarding the imaging parameters included, the thresholds used to define remission, and the role of concomitant endoscopic remission [11]. As a result, current definitions remain largely modality-specific, reflecting the different capabilities of IUS, MRE, and CTE in assessing bowel wall and extramural disease. Understanding how TH is defined and assessed across these imaging techniques is therefore essential for interpreting the available evidence and comparing results among studies.

### 4.1. Intestinal Ultrasound

Among the available cross-sectional imaging modalities, IUS has emerged as one of the most attractive tools for assessing TH in CD because of its non-invasive nature, low cost, bedside availability, and suitability for repeated monitoring. These characteristics make IUS particularly appealing within treat-to-target strategies, where frequent reassessment of disease activity is required [16,17].

Although a universally accepted ultrasound-based definition of TH is still lacking, most studies rely on a common set of sonographic parameters reflecting resolution of bowel wall inflammation. BWT remains the cornerstone of assessment, with normalization generally defined as a thickness ≤ 3 mm in previously affected bowel segments [16,17]. Additional criteria typically include the absence of color Doppler signal (CDS), indicating resolution of mural hypervascularization, preservation or restoration of normal bowel wall stratification (BWS) and the disappearance of inflammatory mesenteric fat and other inflammatory complications such as phlegmons, abscesses, or fistulas [16,18,19]. In most contemporary studies, TH is defined as the normalization of all these parameters rather than BWT alone [11,20,21,22].

Beyond the assessment of remission, IUS has also contributed to the development of the concept of transmural response, which describes a substantial but incomplete improvement in bowel wall inflammation. While definitions vary among studies, transmural response is generally based on a significant reduction in BWT, often accompanied by improvement in CDS and other inflammatory features [11]. This distinction is clinically relevant since partial transmural improvement may still be associated with favorable long-term outcomes [23].

Several scoring systems have been proposed to standardize ultrasound assessment. Composite indices such as the International Bowel Ultrasound Segmental Activity Score (IBUS-SAS), the Bowel Ultrasound Score (BUSS), and the Simple Ultrasound Score for Crohn’s Disease (SUS-CD) integrate key sonographic variables including BWT, CDS, BWS and mesenteric inflammatory changes [24,25,26]. Although these scores were primarily developed to quantify disease activity rather than define TH, they represent important steps toward a more objective and reproducible evaluation of transmural inflammation [24,25,26]. Despite the growing evidence supporting IUS-guided assessment of TH, significant heterogeneity persists regarding the parameters included, the thresholds adopted, and the relative importance assigned to individual sonographic findings. Ongoing international efforts aimed at standardizing definitions of transmural response, transmural remission, and TH are expected to improve comparability among studies and facilitate the integration of ultrasound-defined TH into future clinical trials and routine clinical practice [27].

### 4.2. Magnetic Resonance Enterography

MRE represents a comprehensive cross-sectional imaging modality for the assessment of transmural inflammation in CD. While IUS is increasingly adopted as the first-line imaging tool for longitudinal monitoring of TH because of its bedside availability and repeatability, MRE remains complementary and particularly valuable in patients with disease locations that are difficult to assess sonographically, such as the proximal small bowel, as well as in those with obesity, excessive bowel gas, or other factors limiting ultrasound evaluation [28]. Moreover, MRE provides a detailed assessment of transmural and extramural disease manifestations, including strictures, fistulas, abscesses and mesenteric inflammation [29]. As with IUS, no universally accepted MRE-based definition of TH currently exists. Definitions of transmural healing remain heterogeneous across studies. Although normalization of BWT (most commonly ≤3 mm) represents the core criterion in most definitions, additional imaging features including the absence of mural edema, hyperenhancement, diffusion restriction, extraintestinal inflammatory signs, and penetrating complications have been incorporated inconsistently, highlighting the current lack of a standardized definition [11]. However, some investigators consider radiological remission alone sufficient, whereas others require the concomitant achievement of endoscopic MH [11].

Several MRI activity indices have been developed to quantify transmural inflammation, including the Magnetic Resonance Index of Activity (MaRIA), its simplified version (sMaRIA), and the Pediatric Inflammatory Crohn’s Magnetic Resonance Enterography Index (PICMI) score, a validated MRE-based index for assessing transmural inflammation in pediatric CD [30,31]. More recently, dedicated transmural healing scores such as the MRI-based C-score have been proposed to standardize the assessment of deep remission [32]. Beyond its diagnostic role, MRE has provided some of the strongest evidence linking TH to improved long-term outcomes, including lower rates of hospitalization, surgery, treatment escalation, and bowel damage progression. Nevertheless, variability in imaging criteria and scoring systems continues to limit the standardization of MRE-defined TH across studies [30].

### 4.3. Computed Tomography Enterography

CTE was among the first cross-sectional imaging techniques used to assess transmural inflammation in CD and has contributed to the development of the concept of TH. Similar to MRE, CTE evaluates the entire bowel wall and surrounding mesentery, allowing for the assessment of BWT, mural hyperenhancement, mesenteric inflammatory changes, and penetrating complications such as fistulas, abscesses, and strictures [33]. Although no standardized CTE-based definition of TH exists, most studies have adopted criteria largely overlapping with those used for MRE, including normalization of BWT (typically ≤3 mm), absence of mural enhancement, and resolution of transmural and extramural inflammatory findings [30,33]. Despite its high diagnostic accuracy, the role of CTE in the assessment of TH remains limited by exposure to ionizing radiation, particularly in young patients requiring repeated imaging over time [30,33]. Consequently, current practice generally favors IUS or MRE for longitudinal monitoring, reserving CTE for selected clinical scenarios in which these modalities are unavailable, contraindicated, or technically inadequate [11,30].

Overall, IUS and MRE should be regarded as complementary rather than competing imaging modalities for the assessment of TH in CD. IUS is particularly suited for tight longitudinal monitoring because of its bedside availability, low cost, and repeatability, whereas MRE is preferred for the comprehensive assessment of proximal small bowel disease, penetrating complications, and situations in which ultrasound evaluation is technically limited. CTE retains a role in selected clinical scenarios but is less suitable for serial monitoring because of cumulative radiation exposure [11,30] (Table 1).

### 4.4. Emerging Technologies

Although BWT and vascularity remain the cornerstone of current TH assessment, several emerging imaging technologies may further refine the evaluation of deep remission in CD. Among IUS-based techniques, contrast-enhanced ultrasound (CEUS) may provide detailed characterization of bowel wall vascularity and perfusion, improving the detection of residual inflammatory activity [34,35]. Similarly, elastography represents another promising adjunctive technique. By measuring bowel wall stiffness, both strain and shear-wave elastography provide a non-invasive estimate of intestinal fibrosis, a key factor limiting the achievement of TH. Preliminary studies suggest that increased bowel stiffness is associated with a lower likelihood of achieving TH and a higher risk of surgery, highlighting the potential role of elastography in distinguishing inflammatory from fibrostenotic disease [36].

More recently, radiomics and artificial intelligence (AI) have emerged as potential tools for the quantitative assessment of transmural disease activity. Machine learning models applied to MRE and CTE can extract imaging features not detectable by visual inspection alone, showing promising performance in predicting both MH and TH [37]. Novel MRI-based scores such as the machine-learning-derived SYSU score have demonstrated encouraging results in predicting long-term outcomes and bowel damage progression [38].

Finally, AI-assisted image analysis may improve the reproducibility of both IUS and MRE by automating bowel wall segmentation, thickness measurements, and feature extraction. Although these technologies remain largely investigational, they may contribute to the development of more objective, standardized, and outcome-driven definitions of TH, ultimately facilitating its integration into precision treat-to-target strategies [39].

## 5. Is Transmural Healing Achievable with Current Therapies in Crohn’s Disease?

### 5.1. Evidence Across Biologics and Small Molecules

- Anti-TNFalpha

Anti-TNF agents, specifically infliximab (IFX) and adalimumab (ADA), provide the most robust evidence regarding the achievability and clinical relevance of TH in CD. Early evidence came from the prospective study by Castiglione et al., who demonstrated that TH, assessed by IUS after two years of therapy, could be achieved in approximately 25% of patients treated with anti-TNF agents compared with only 4% of those receiving thiopurines (OR 6.2; *p* < 0.01). Importantly, TH showed a strong association with MH, suggesting that resolution of bowel wall inflammation represents a deeper level of disease control beyond MH [40]. Subsequent studies confirmed these findings in larger cohorts. In the prospective multicenter study by Calabrese et al., including 188 patients treated with different biologic agents, TH was achieved after 12 months in 37% of patients receiving IFX and 26.8% of those receiving ADA. Notably, greater baseline BWT was independently associated with a lower probability of achieving TH at both 3 months (OR 0.70, 95% CI 0.50–0.97) and 12 months (OR 0.58, 95% CI 0.38–0.89), whereas shorter disease duration favored the achievement of deep healing [22]. In a prospective study including 218 patients treated with IFX or ADA for two years, Castiglione et al. defined TH as normalization of BWT (≤3 mm) on IUS and reported TH in 68 of 218 patients (31.2%), compared with 27.5% achieving MH alone and 41.3% showing no healing [9]. Evidence from cross-sectional imaging studies has further supported the efficacy of anti-TNF therapy in achieving deeper healing targets. In the study by Oh et al., involving 392 patients treated with anti-TNF agents, radiologic healing was defined as BWT < 3 mm, absence of mural hyperenhancement, normalization of mural signal intensity, absence of perienteric inflammation, and no worsening stricturing or penetrating complications on MRE or CTE. Using these criteria, 29.1% of patients achieved combined MH and radiologic healing, whereas 15.0% achieved MH alone and 10.4% radiologic healing alone [6]. Collectively, these studies suggest that anti-TNF therapy can induce transmural healing in approximately 25–35% of patients, although rates vary according to disease duration, imaging modality, and the definition of TH adopted.

- Vedolizumab

The most robust data regarding TH with vedolizumab (VDZ) are derived from the VERSIFY study, a prospective phase 3b trial evaluating endoscopic, radiologic, and histologic healing in 101 patients with moderately to severely active CD, more than half of whom had previously failed anti-TNF therapy. Radiologic healing, defined as a MaRIA score < 7 on MRE, was achieved in 21.9% of patients at week 26 and increased to 38.1% at week 52. Interestingly, radiologic healing rates exceeded MH rates (11.9% and 17.9% at weeks 26 and 52, respectively), suggesting that cross-sectional imaging may capture deeper therapeutic responses not fully reflected by endoscopic assessment alone [41]. Further insights were provided by a post hoc MRE substudy of VERSIFY, which included 27 patients with evaluable imaging at week 26 and 13 patients followed through week 52. This analysis demonstrated a progressive reduction in individual markers of transmural inflammation, including bowel wall edema and BWT, supporting the concept that TH may continue to evolve beyond the induction phase and requires prolonged therapeutic exposure [42]. Real-world studies have yielded comparable results. In the multicenter prospective study by Calabrese et al., where IUS-assessed TH was defined as normalization of all ultrasonographic parameters, after one year of treatment, TH was achieved in 27.2% of VDZ-treated patients [22]. Similarly, the prospective SUNRISE study enrolled 70 patients with moderately to severely active CD initiating VDZ therapy and monitored disease activity using serial IUS. At six months, TH, defined as normalization of BWT (≤3 mm), was achieved in 9 of 28 evaluable patients (32.1%), while transmural response, reflecting substantial IUS improvement without complete normalization, was observed in 12 of 28 patients (42.9%). Notably, SUNRISE incorporated TH remission into a composite endpoint of complete remission, requiring the simultaneous achievement of corticosteroid-free clinical remission and biomarker normalization as well. Although follow-up remains limited and formal comparative analyses are not yet available, these findings provide prospective real-world evidence supporting the ability of VDZ to induce deep transmural improvement [43]. Finally, the ongoing VECTORS trial represents the first prospective treat-to-target study to incorporate IUS-defined transmural healing as part of the therapeutic strategy. In this study, TH is defined as BWT ≤ 3 mm and complete normalization of CDS in all evaluable bowel segments. Although mature efficacy data are not yet available, preliminary results have shown substantial reductions in BWT and ultrasound activity scores within the first 14 weeks of treatment, highlighting the feasibility of integrating transmural endpoints into routine disease monitoring [44].

- Ustekinumab

One of the largest real-world experiences on ustekinumab (UST) efficacy in inducing TH was reported by Miranda et al., who prospectively evaluated 92 patients with moderate-to-severe CD previously exposed to conventional therapies and/or biologics. TH was assessed by MRE or IUS after 52 weeks of treatment. Among the 75 patients who completed follow-up, MH was observed in 35% of patients, while not all patients achieving MH also fulfilled criteria for TH, with a TH rate of 20%. These findings suggest that although UST is effective in inducing clinical and endoscopic improvement, complete deep remission remains challenging to achieve in a population largely composed of biologic-experienced patients [45]. Additional evidence derives from the multicenter prospective study by Calabrese et al., in which, among 30 UST-treated patients, TH was achieved in 20.0% after one year of therapy, representing the lowest rate among the biologic classes evaluated in the study. Notably, patients treated with IFX (37.0%) and ADA (26.5%) were significantly more likely to achieve TH compared with those receiving UST (IFX vs. UST: HR 2.7, 95% CI 1.9–6.4, *p* = 0.017; ADA vs. USTE: HR 2.1, 95% CI 1.12–3.9, *p* = 0.02) [22]. The most detailed assessment of transmural outcomes with UST comes from the IUS substudy of the phase 3b STARDUST trial. Seventy-seven patients underwent serial IUS examinations, with TH defined by normalization of BWT, CDS, BWS, and inflammatory mesenteric fat. IUS response was detectable as early as week 4 and progressively increased throughout follow-up. At week 48, TH was achieved in 24.1% of patients, while 46.3% fulfilled criteria for IUS response [20].

Furthermore, MRI-based studies have been conducted in this setting. In the retrospective cohort by Lafeuille et al., which defined TH as the combination of MH and MRI healing, no UST-treated patients achieved TH, although the number of exposed patients was very small [8]. More recently, Revés et al. reported MRI-defined TH in 4 of 13 UST-treated patients (30.8%), highlighting the substantial variability that currently exists across studies because of differences in patient populations, imaging modalities, and healing definitions [10].

Overall, the available evidence suggests that UST induces TH in approximately 20–30% of treated patients. Although these rates appear somewhat lower than those reported with anti-TNF agents, comparisons across studies should be interpreted cautiously given the heterogeneity of definitions and the frequent inclusion of highly refractory, biologic-experienced populations in UST cohorts.

- Selective IL-23 inhibitors

Evidence regarding TH with risankizumab (RISA) is still limited but rapidly expanding. Available real-world studies suggest that selective IL-23 blockade induces substantial transmural improvement, although complete TH remains challenging to achieve, particularly in highly refractory populations [21,46].

The largest real-world experience was reported by Scaldaferri et al., who evaluated 520 patients initiating RISA. This represented a particularly difficult-to-treat population, with 45% of patients having failed at least three advanced therapies and 54.8% previously exposed to UST. In the study, radiologic remission, defined as BWT ≤ 4 mm in the absence of mural hyperenhancement/hyperemia and without stricturing or penetrating complications, occurred in 25.7% and 24.6% of patients at weeks 26 and 52, respectively. In contrast, the more stringent endpoint of TH, requiring a normalized BWT ≤ 3 mm together with the same ancillary criteria, was achieved in 13.9% of patients at week 26 and 9.8% at week 52. Despite these relatively modest TH rates, imaging demonstrated significant structural improvement, with mean BWT decreasing from 7.2 mm at baseline to 5.6 mm at week 52 (*p* < 0.001) and cumulative diseased bowel length decreasing from 249 mm to 75 mm (*p* = 0.018) [21].

The kinetics of transmural response were prospectively investigated by Domas et al. in a multicenter cohort of 101 patients undergoing serial IUS assessments [46]. Transmural response was defined as a ≥25% reduction in the IBUS-SAS, whereas TH required complete normalization of IUS findings. Importantly, transmural response was already detectable after one month of treatment, occurring in 27.3% of patients at week 4 and 28.5% at week 12. Nevertheless, complete TH remained uncommon, being achieved in only 8.5% of patients at week 12. Together, these findings suggest that RISA induces early transmural improvement, whereas complete bowel wall normalization likely requires longer treatment exposure [46].

Additional IUS-based evidence comes from the prospective study by Rosentreter et al., which evaluated 33 patients undergoing serial IUS examinations after RISA initiation [47]. Ultrasound response was defined as a ≥25% reduction in BWT, while TH required normalization of BWT, CDS, BWS and inflammatory fat. Among patients with colonic disease, 80% achieved an IUS response at 3 months, accompanied by a significant reduction in mean BWT from 5.6 mm to 3.5 mm (*p* = 0.011). Conversely, only 20% of patients with ileal disease achieved an IUS response at 3 months and 21.4% at 6 months. Although fecal calprotectin significantly decreased both at 3 months (*p* = 0.008) and 6 months (*p* = 0.02), TH was achieved in only one patient during follow-up. While limited by its small sample size, this study further supports the notion that RISA may induce measurable transmural improvement before complete TH becomes evident [47]. Further support comes from a recent Taiwanese multicenter cohort reported by Chung et al., including 49 patients with moderate-to-severe CD. Notably, TH was documented in 16.3% of patients after only 12 weeks of therapy [48].

Overall, current evidence suggests that RISA is effective in inducing substantial transmural improvement in CD. However, complete TH remains relatively uncommon during the first year of therapy, with reported rates generally ranging between 10% and 20%. Nevertheless, this data should be interpreted cautiously given the inclusion of highly refractory, advanced therapies-experienced populations in the analyzed cohorts.

Whether deeper and more sustained suppression of the selective IL-23 pathway can further increase TH rates remains an open question. In this regard, the ongoing REASON trial with guselkumab (NCT06408935), which incorporates MRE-defined TH assessed through the MaRIA score as a key outcome at week 48, may provide important insights into the potential of selective IL-23 inhibition to achieve TH in CD [49].

- Upadacitinib

Upadacitinib, a selective Janus kinase 1 (JAK1) inhibitor, represents the first approved JAK inhibitor for CD with accumulating real-world evidence indicates that JAK1 inhibition may promote rapid transmural improvement, including bowel wall normalization in a subset of patients with highly refractory disease. The most detailed assessment of transmural outcomes was provided by Bezzio et al. in a multicenter Italian cohort of 64 patients with moderate-to-severe CD treated through a compassionate-use program [50]. Notably, all patients had previously failed all currently reimbursed advanced therapies, including anti-TNF agents, VEDO, and UST, while two-thirds had already undergone intestinal surgery. Transmural response was defined as a reduction in BWT of at least 2 mm, whereas TH required normalization of BWT below 3 mm on IUS. After 12 weeks of treatment. Ultrasound assessment demonstrated transmural response in 73.0% of patients and TH in 15 of 52 evaluable subjects (28.8%). Importantly, deep remission, defined as the simultaneous achievement of clinical remission, biochemical remission, and TH, was observed in 21.8% of the entire cohort. Although coming from a retrospective study, these findings are particularly noteworthy considering the highly refractory nature of the study population and suggest that UPA may induce substantial bowel wall improvement within a relatively short period of treatment [50].

Additional real-world evidence was reported by Wu et al. in a multicenter retrospective cohort of 156 patients with CD treated with UPA. At week 12, imaging outcomes revealed a discrepancy between response and complete remission. Radiological evaluation with MRE or CTE demonstrated improvements in remission rates from 1.1% (1/91) to 9.1% (2/22) (*p* < 0.001), with 85.7% (18/21) achieving radiological response at week 12. These findings suggest that although complete normalization of bowel wall abnormalities remains relatively uncommon during short-term follow-up, a substantial proportion of patients experience meaningful transmural improvement [51].

Further insights were provided by Xie et al., who specifically investigated the efficacy of UPA in patients with isolated small bowel CD, a phenotype traditionally considered more difficult to treat and less likely to achieve deep healing. In this retrospective cohort of 98 patients receiving 12 weeks of induction therapy, TH rates were comparable between patients with isolated small bowel disease and those with non-small bowel involvement (30.0% vs. 36.2%). Both groups showed significant reductions in IBUS-SAS activity scores and CDAI from baseline, supporting the effectiveness of UPA across different disease locations. Importantly, multivariable analysis identified previous intestinal surgery as an independent negative predictor of radiological remission (OR 0.287, 95% CI 0.094–0.877; *p* = 0.029), suggesting that cumulative structural bowel damage may limit the likelihood of achieving transmural endpoints despite adequate suppression of inflammation [52].

Although the available evidence consistently points toward a favorable effect of UPA on transmural outcomes, the current literature remains limited by its predominantly retrospective design and the absence of prospective studies specifically powered to evaluate TH. Consequently, estimates of transmural response and TH should be considered exploratory. Across studies, transmural response rates frequently exceeded 50–70% within the first months of therapy, whereas complete TH was achieved in approximately 10–30% of patients, depending on the definition and imaging modality adopted. These findings highlights the need for standardized definitions and prospective validation of transmural endpoints in patients receiving JAK inhibition (Table 2).

### 5.2. Factors Associated with Achieving Transmural Healing

Although TH can be achieved with currently available therapies, not all patients appear equally likely to reach this ambitious endpoint. Several studies have identified clinical, disease-related, and treatment-related factors associated with a higher probability of achieving TH [10,22,53].

One of the most consistent predictors is early intervention. In a multicenter MRE-based study, Revés et al. demonstrated that initiation of biologic therapy within 12 months of diagnosis was independently associated with a more-than-threefold-increased likelihood of achieving TH (aOR 3.23, 95% CI 1.36–7.70). These findings support the concept that early suppression of inflammation may prevent the development of irreversible structural bowel damage and increase the likelihood of achieving deep tissue healing, suggesting the presence of a “window of opportunity” during which timely therapeutic intervention may maximize the probability of achieving TH and long-term disease modification [10].

Disease severity at baseline also appears to influence the probability of TH. In a multicenter study by Calabrese et al., greater BWT at IUS baseline before treatment initiation was associated with a lower probability of achieving TH at 3 months [*p* = 0.03 (OR 0.70, 95% CI 0.50–0.97)] and 12 months [*p* = 0.01 (OR0.58,95%CI 0.38–0.89)] [22]. Similarly, Maconi et al. observed that patients achieving TH during long-term anti-TNF therapy had lower baseline BWT than those with persistent transmural inflammation, suggesting that advanced bowel wall damage may reduce the reversibility of transmural lesions [53].

Beyond inflammatory burden, disease phenotype may also play a critical role in determining the likelihood of achieving TH. In the study by Maconi et al., patients with a stricturing phenotype were significantly less likely to achieve a transmural response. Notably, stenosing disease was present in 85% of non-responders compared with 43% of responders, and stricturing behavior remained independently associated with the absence of transmural response in multivariable analysis (*p* = 0.002). In contrast, shorter disease duration and a non-stricturing phenotype were associated with more favorable transmural outcomes. These findings suggest that established fibrostenotic damage may represent a major obstacle to achieving complete bowel wall healing, even when inflammatory activity is adequately controlled [53].

Treatment-related factors may further influence the achievement of TH. Maconi et al. also demonstrated that lower anti-TNF trough levels were associated with persistent bowel wall inflammation, with significantly lower serum concentrations observed among non-responders (overall anti-TNF levels, *p* = 0.005). In multivariable analysis, low anti-TNF drug levels independently predicted the absence of transmural response (*p* = 0.01), supporting a potential role for therapeutic drug monitoring as a strategy to optimize deep healing outcomes [53].

In addition, emerging data suggest that previous biologic exposure may influence the probability of achieving transmural endpoints. In the large real-world cohort reported by Scaldaferri et al., patients treated with RISA who were naïve to UST achieved significantly higher rates of radiologic remission (43.5% vs. 13.2%, *p* = 0.008) and TH (26.1% vs. 0%, *p* = 0.001) compared with UST-exposed patients, suggesting that earlier positioning of advanced therapies within the treatment algorithm may improve the likelihood of achieving deep remission [21].

Beyond baseline predictors, early treatment response may itself provide important prognostic information. In the STARDUST ultrasound substudy, outcomes were more favorable among biologic-naïve patients and those with colonic disease. Notably, the absence of an ultrasound response as early as week 4 was associated with failure to achieve endoscopic response at week 48, with a negative predictive value of 73% [20]. Interestingly, a potential influence of disease location on transmural outcomes has also been suggested by other ultrasound-based studies. In the RISA cohort, Rosentreter et al. reported substantially higher transmural response rates in colonic compared with ileal disease (80% vs. 20% at 3 months), supporting the hypothesis that colonic involvement may be more amenable to TH than isolated ileal disease [47].

**Figure 1:**
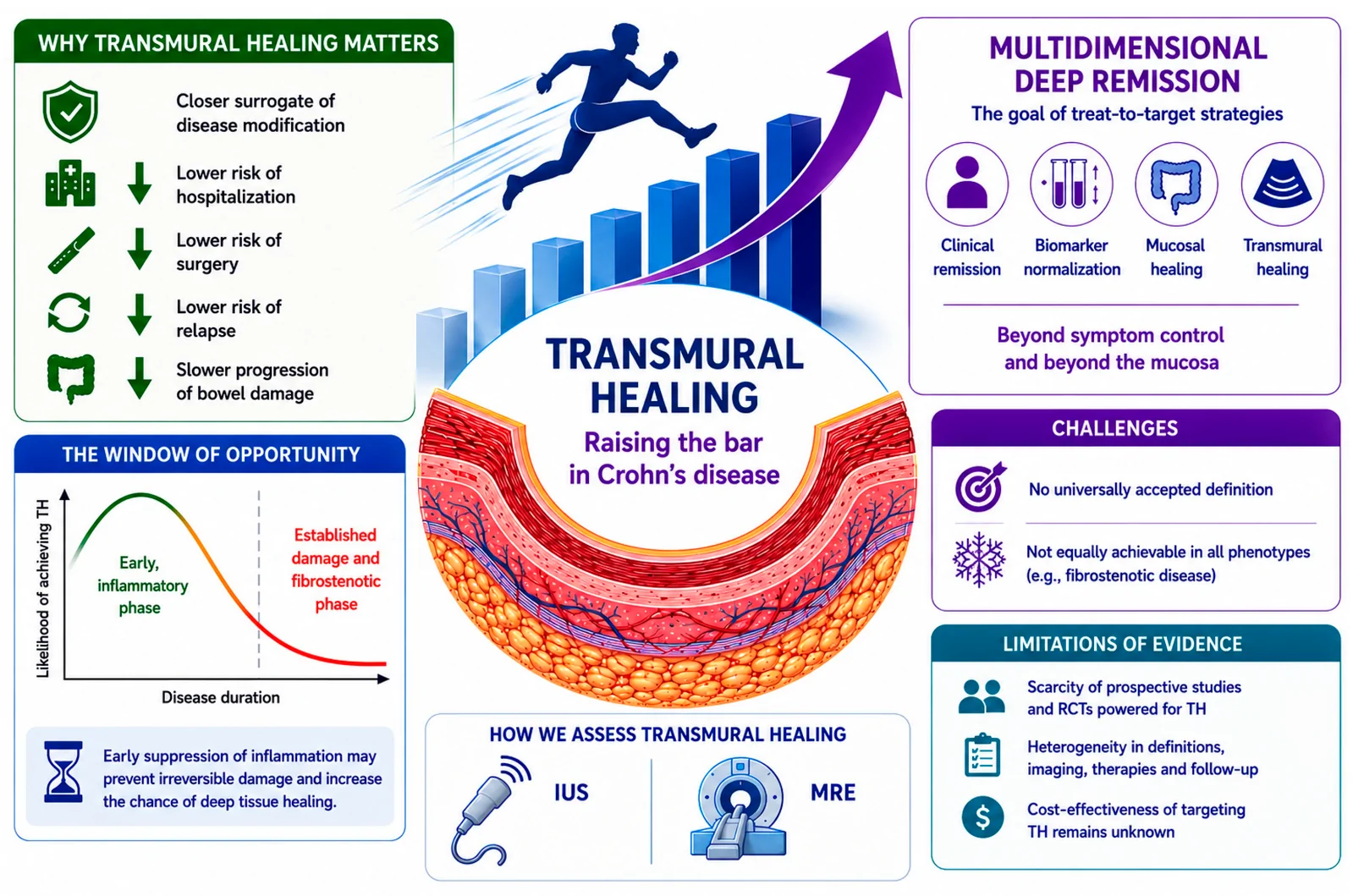
Raising the Bar in Crohn’s Disease: Transmural Healing as a Step Toward Multidimensional Deep Remission.

Although these findings require confirmation in larger cohorts, they collectively suggest that IUS may serve not only as a tool for assessing TH, but also as an early biomarker of treatment effectiveness and a means of identifying patient subgroups more likely to achieve deep remission. Taken together, current evidence suggests that TH is more frequently achieved in patients with early, predominantly inflammatory disease, limited structural bowel damage, non-stricturing behavior, lower baseline BWT, adequate biologic exposure, and possibly earlier use of advanced therapies [20,21,47,53]. However, the predictors of TH remain incompletely characterized, and the available evidence is largely derived from heterogeneous studies with different imaging modalities, definitions of TH, treatment strategies, and follow-up durations. Further prospective studies are therefore needed to validate these candidate predictors and to establish reliable models for identifying patients most likely to achieve TH (Figure 1).

## 6. Discussion

TH has emerged as one of the most promising therapeutic concepts in CD, reflecting the growing recognition that a disease characterized by transmural inflammation cannot be fully captured by mucosal assessment alone [11]. Persistent bowel wall inflammation, progressive structural damage, and ongoing extramural disease activity may occur despite apparent MH, highlighting the limitations of a purely mucosal paradigm and supporting a shift toward therapeutic targets that better reflect the full burden of disease. The prognostic value of TH is now supported by a growing body of evidence demonstrating superior long-term outcomes compared with MH alone [5,6,9]. These findings suggest that TH may represent a closer surrogate of true disease modification than MH and provide a strong biological rationale for its consideration as a therapeutic target.

However, several important barriers currently limit its integration into routine clinical practice and formal treatment algorithms. The most important challenge remains the absence of a universally accepted definition. While normalization of BWT is the most consistently adopted criterion across studies, substantial heterogeneity persists and consequently, reported TH rates vary considerably across studies and imaging modalities, making direct comparisons difficult [11,27]. Recent advances, including ongoing standardization initiatives, represent important steps forward. In particular, the ongoing TRENCH-1 study (NCT05903066), a prospective multicenter study specifically designed to develop an objective and reproducible MRE-based definition of TH, is expected to provide a structured grading system for the assessment of TH and to evaluate the reproducibility of radiological findings across observers. Its results may contribute significantly to the standardization of TH assessment in both clinical practice and future clinical trials [54]. In the context of IUS, establishing a universally accepted definition of TH should rely on international consensus efforts led by scientific societies such as ECCO and IBUS. The widespread implementation of TH as a treatment target will require harmonized scanning protocols, standardized and validated IUS scoring systems, and prospectively validated thresholds associated with long-term clinical outcomes.

Beyond standardization, an equally important challenge is whether current therapeutic strategies are sufficient to achieve TH in a substantial proportion of patients. Achievement of TH remains limited and inconsistent across therapeutic classes with a systematic review of 17 studies reporting TH rates ranging from 14.7% to 42.7% [11]. However, interpretation of these data is complicated by the relative scarcity of prospective studies specifically designed around transmural endpoints. Most available evidence derives from observational cohorts, retrospective analyses, or post hoc evaluations, whereas randomized controlled trials powered to assess TH remain limited. These study suggest the concept of a therapeutic window of opportunity, supporting that earlier intervention, before the development of irreversible bowel damage, may maximize the likelihood of achieving deep tissue healing and ultimately disease modification. Ongoing prospective studies such as VECTORS and REASON will provide important insights into the feasibility of incorporating transmural endpoints into treat-to-target strategies and may clarify whether actively pursuing TH translates into improved long-term outcomes [44,49].

At the same time, the limited rates of TH observed across therapeutic classes underscore the persistent challenge of fibrosis, which remains one of the major unmet needs in CD. Even when inflammation is effectively controlled, established fibrostenotic changes may prevent complete restoration of bowel wall architecture, emphasizing the need for imaging tools capable of distinguishing inflammatory from fibrotic damage and for therapies specifically targeting fibrogenesis. Future therapeutic advances will therefore need not only to improve inflammatory control but also to address the structural component of the disease, potentially increasing the proportion of patients able to achieve TH [55].

The persistent challenge of fibrosis may also help explain why TH remains difficult to achieve despite effective control of inflammation [11]. Consequently, the distinction between transmural response and complete TH becomes increasingly relevant. While TH represents the deepest level of tissue healing, transmural response, reflecting a substantial reduction in transmural inflammation without complete normalization, may represent a more attainable and clinically meaningful endpoint, particularly in patients with established structural bowel damage. Emerging evidence suggests that outcomes improve along a continuum from persistent transmural inflammation to transmural response and ultimately TH, supporting the concept that TH is not a binary phenomenon but a progressive process of tissue repair. From a treat-to-target perspective, transmural response serves as an intermediate target and an early indicator of treatment success, as part of sequential therapeutic milestones [11,55]. In this context, IUS may be uniquely positioned to monitor this stepwise healing process and to facilitate the incorporation of transmural targets into routine treat-to-target strategies, given its ability to provide repeated, non-invasive assessment of transmural disease activity over time [17].

Unlike previous reviews, this work not only summarizes the available evidence on TH, but also critically discusses the current challenges related to its definition, compares the strengths and limitations of the available imaging modalities, and highlights the evolving role of IUS within treat-to-target strategies and future therapeutic algorithms.

Looking ahead, advances in AI, radiomics, and quantitative imaging analysis may substantially improve the reproducibility and standardization of transmural assessment [39]. Beyond technical innovations, future therapeutic strategies in CD are likely to move toward multidimensional definitions of deep remission integrating clinical remission, biomarker normalization, MH, and TH into a comprehensive measure of disease control. Future research should also establish the optimal timing and frequency of imaging reassessment and determine the cost effectiveness of routine transmural monitoring in different healthcare settings. Whether this approach will ultimately translate into true disease modification remains to be established. As definitions become standardized and therapeutic strategies continue to evolve, TH appears increasingly positioned not merely as an imaging endpoint, but as a potential marker of disease modification. In this context, TH may represent the next major step in the evolution of treat-to-target strategies, effectively raising the bar in CD.

## 7. Conclusions

TH has emerged as one of the most promising therapeutic concepts in CD, reflecting the shift from symptom control toward objective measures of disease modification. However, despite its strong biological rationale and growing clinical relevance, TH cannot yet be considered an established treatment target because of the lack of standardized imaging definitions, the heterogeneity of assessment across imaging modalities, and the absence of prospective treat-to-target trials specifically evaluating TH-guided management.

Future efforts should focus on harmonizing TH definitions and imaging protocols, validating reproducible and clinically meaningful thresholds, and determining the optimal timing of imaging reassessment. In addition, randomized studies are needed to establish whether treatment strategies targeting TH improve long-term outcomes beyond current endoscopic-based approaches and whether they are cost-effective in routine clinical practice. As these challenges are addressed, TH has the potential to evolve from a promising imaging endpoint into a validated therapeutic target, further advancing treat-to-target strategies and bringing clinical practice closer to the goal of durable disease modification in CD.

## Figures and Tables

**Table 1:** Advantages and limitations of imaging modalities for TH assessment in Crohn’s disease.

Modality	Core TH Criteria (Most Commonly Used)	Main Scores	Advantages	Main Limitations
IUS	BWT ≤ 3 mm, absent Doppler signal, restoration of BWS, resolution of inflammatory fat	IBUS-SAS BUSS SUS-CD	Bedside, repeatable, inexpensive, suitable for tight monitoring	Operator dependency, limited assessment of deep pelvic/proximal small bowel disease, lack of standardized TH definition
MRE	BWT ≤ 3 mm, absence of mural edema, ulcers, contrast enhancement, diffusion restriction and inflammatory complications	MaRIA sMaRIA PICMI C-score	Comprehensive bowel wall and extramural assessment, reproducible	Cost, accessibility, heterogeneity of TH definitions
CTE	BWT normalization, absence of mural hyperenhancement and inflammatory complications	No validated TH-specific score	Wide availability, rapid acquisition	Radiation exposure, limited suitability for repeated monitoring

Abbreviations: TH, transmural healing; IUS, intestinal ultrasound; BWT, bowel wall thickness; BWS, bowel wall stratification; IBUS-SAS, International Bowel Ultrasound Segmental Activity Score; BUSS, Bowel Ultrasound Score; SUS-CD, Simple Ultrasound Score for Crohn’s Disease; MRE, magnetic resonance enterography; MaRIA, Magnetic Resonance Index of Activity; sMaRIA, simplified Magnetic Resonance Index of Activity; PICMI, Pediatric Inflammatory Crohn’s MRE Index; CTE, computed tomography enterography.

**Table 2:** Selected evidence on transmural healing across therapeutic classes and imaging modalities in Crohn’s disease.

Therapy	Study	Design	N	Imaging Modality	Definition of TH	Follow-Up	TH Rate
**Anti-TNF**	Castiglione, 2013 [40]	Prospective longitudinal	66	IUS	BWT ≤ 3 mm	24 mo	TH: 17/66 (25.8%)
	Castiglione, 2019 [9]	Prospective longitudinal	218	IUS	BWT ≤ 3 mm in all inflamed segments	24 mo	TH: 68/218 (31.2%)
	Calabrese, 2022 [22]	Prospective multicenter	188	IUS	Normalization of all IUS parameters	12 mo	IFX: TH: 11/31 (37.0%); ADA: TH: 27/103 (26.8%)
	Oh, 2022 [6]	Retrospective single-center	392	MRE/CTE + endoscopy	BWT < 3 mm, absence of mural hyperenhancement, normal mural signal, no perienteric infiltration, absence of newly developed or worsening preexisting stricturing or penetrating complications	>12 mo	MH + TH: 114/392 (29.1%) TH only: 41/392 (10.4%)
	Lafeuille, 2021 [8]	Retrospective cohort	154	MRI + endoscopy	MRI-H + MH	Median FU: 29.1 mo	IFX: TH: 5/28 (17.9%) MRI-H: 6/30 (20.0%); ADA: TH:4/28 (14.3%) MRI-H: 2/30 (6.7%)
	Revés, 2025 [10]	Multicenter, retrospective	154	MRE	Normalization of all MRE parameters	Median FU: 68 mo	IFX: TH: 20/85 (23.5%) ADA: TH: 8/55 (14.5%)
**Vedolizumab**	Rimola, 2024 [42]	Phase 3b prospective trial	101	MRE	MaRIA < 7	26–52 wk	TH week 26: 8/37 (21.9%) TH week 52: 4/22 (38.1%)
	Carter, 2025 [43]	Prospective observational	70	IUS	IUS transmural remission	6 mo	TH: 9/28 (32.1%)
	Calabrese, 2022 [22]	Prospective multicenter	24	IUS	Normalization of all IUS parameters	12 mo	TH: 27.2%
	Lafeuille, 2021 [8]	Retrospective cohort	154	MRI + endoscopy	MRI-H + MH	Median FU: 29.1 mo	VDZ: TH: 0/28 (0.0%) MRI-H: 1/30 (3.3%)
**Ustekinumab**	Miranda, 2021 [45]	Prospective multicenter real-world	92	MRI/IUS	MRI: Normalization of all parameters; IUS: BWT < 3 mm and normal IUS examination	52 wk	TH: 15/75 (20.0%)
	Kucharzik, 2023 [20]	Phase 3b RCT	77	IUS	Normalization of BWT, CDS, BWS, i-fat	48 wk	TH: 13/54 (24.1%)
	Calabrese 2022 [22]	Prospective multicenter	188	IUS	Normalization of all IUS parameters	12 mo	6/30 (20%)
	Lafeuille, 2021 [8]	Retrospective cohort	154	MRI + endoscopy	MRI-H + MH	Median FU: 29.1 mo	UST: TH: 0/28 (0%) MRI-H: 0/30 (0%)
	Revés, 2025 [10]	Multicenter, retrospective	154	MRE	Normalization of all MRE parameters	Median FU: 68 mo	TH: 4/13 (30.8%)
**Risankizumab**	Scaldaferri, 2026 [21]	Multicenter retrospective cohort	520	Cross-sectional imaging	BWT ≤ 3 mm + no hyperemia/enhancement + no complications	52 wk	TH: 51/520 (9.8%)
	Domas, 2026 [46]	Prospective multicenter	101	IUS	IUS parameters normalization	12 wk	TH: 8.5%
	Chung, 2025 [48]	Multicenter retrospective cohort	49	Cross-sectional imaging	IUS: BWT < 3 mm by IUS MRE/CTE: absence of transmural inflammation	12 wk	TH: 8/49 (16.3%)
	Rosentreter, 2024 [47]	Prospective observational	33	IUS	Normalization of BWT and CDS	6 mo	TH: 1/18 (5.6%)
**Upadacitinib**	Bezzio, 2024 [50]	Observational retrospective cohort	64	IUS	BWT < 3 mm	12 wk	TH: 15/52 (28.8%)
	Wu, 2025 [51]	Multicenter retrospective cohort	156	MRE/CTE	Normalization of BWT, inflammatory fat, mural blood flow, and enhancement	12 wk	TH: 2/22 (9.1%)
	Xie et al., 2026 [52]	Retrospective cohort	98	IUS	IBUS-SAS normalization	12 wk	TH: ISB-CD: 12/40 (30.0%); NISB-CD: 21/58 (36.2%)

Direct comparisons across therapies should be interpreted cautiously because definitions of TH, imaging modalities, disease duration, and follow-up schedules differed substantially among studies. Abbreviations: CD: Crohn’s Disease, IUS: intestinal ultrasound; MRE: magnetic resonance enterography; CTE: computed tomography enterography; TH: transmural healing, MH: mucosal healing; MRI-H: MRI-healing; BWT: bowel wall thickness; CDS: color Doppler signal; FU: follow-up; IBUS-SAS: International Bowel Ultrasound Segmental Activity Score; IFX: infliximab; ADA: Adalimumab; VDZ: vedolizumab; UST: ustekinumab; ISB-CD: isolated small bowel CD; NISB-CD: non isolated small bowel CD.

## Data Availability

No new data were created or analyzed in this study. Data sharing is not applicable to this article.

## Abbreviations

The following abbreviations are used in this manuscript:

ADA	Adalimumab
AI	Artificial Intelligence
aHR	Adjusted Hazard Ratio
aOR	Adjusted Odds Ratio
BWS	Bowel Wall Stratification
BWT	Bowel Wall Thickness
BUSS	Bowel Ultrasound Score
CDAI	Crohn’s Disease Activity Index
CD	Crohn’s Disease
CDS	Color Doppler Signal
CEUS	Contrast-Enhanced Ultrasound
CI	Confidence Interval
CTE	Computed Tomography Enterography
HR	Hazard Ratio
IBD	Inflammatory Bowel Disease
IBUS-SAS	International Bowel Ultrasound Segmental Activity Score
IFX	Infliximab
i-fat	Inflammatory Mesenteric Fat
IUS	Intestinal Ultrasound
JAK	Janus Kinase
MaRIA	Magnetic Resonance Index of Activity
MH	Mucosal Healing
MRE	Magnetic Resonance Enterography
MRI	Magnetic Resonance Imaging
OR	Odds Ratio
PICMI	Pediatric Inflammatory Crohn’s MRE Index
RISA	Risankizumab
sMaRIA	Simplified Magnetic Resonance Index of Activity
SUS-CD	Simple Ultrasound Score for Crohn’s Disease
SYSU	Sun Yat-sen University Score
TH	Transmural Healing
TNF	Tumor Necrosis Factor
UPA	Upadacitinib
UST	Ustekinumab
VDZ	Vedolizumab
